# “I was reaching out for help and they did not help me”: Mental healthcare in the carceral state

**DOI:** 10.1186/s40352-022-00183-9

**Published:** 2022-07-25

**Authors:** Anna G. Preston, Alana Rosenberg, Penelope Schlesinger, Kim M. Blankenship

**Affiliations:** 1 Department of Social and Behavioral Sciences, Yale School of Public Health, Yale University, 60 College Street, New Haven, CT 06520 USA; 2 Yale School of Medicine, Yale University, 333 Cedar Street, New Haven, CT USA; 3 Department of Epidemiology of Microbial Diseases, Yale School of Public Health, Yale University, 60 College Street, New Haven, CT 06520 USA; 4 Department of Sociology, American University, 4400 Massachusetts Ave, Washington DC, NW 20016 USA

**Keywords:** Mental health, Carceral state, Incarceration, Racism, Psychiatric medication, Trauma

## Abstract

**Background:**

Despite the limitations the carceral environment may impose on mental wellness, mental healthcare is increasingly becoming a carceral endeavor. Over the course of the last several decades, prisons and jails have become the de facto mental healthcare provider for thousands of incarcerated individuals. Furthermore, practices like mandated mental healthcare for supervised individuals further broaden the population experiencing mental healthcare within the criminal legal system at large. This study examines the perspectives of nine individuals who experienced mental healthcare within the carceral state, whether in prison or on parole or probation, with a special focus on how attributes of the carceral state create ideological and functional barriers to effective mental healthcare.

**Methods:**

Data for the parent study of this analysis was collected via in-depth, one-on-one interviews of about one hour’s length, conducted at six-month intervals over the course of 2 years. These interviews were analyzed using an iterative process of open-coding, thematic code development, and code application to participant interviews.

**Results:**

The results showed a common perception of mental healthcare received within the carceral state as serving goals of the prison system, including control and punishment, rather than therapeutic goals of healing and empowerment. This often had negative implications for the quality of the treatment received, including patterns of diagnostic ambiguity, treatment that was ill-fitting to participants’ needs, and treatment that was undermined by the new trauma created by the prison environment. The results also highlighted racial disparities prevalent within the carceral system. Despite the barriers created by the subjection of therapeutic practices to carceral goals, participants demonstrated resourcefulness and creativity in engaging with these treatment modalities to reap benefits where possible.

**Conclusions:**

Overall, these results highlight the inappropriateness of combining therapeutic and carceral spaces, the need for greater public attention to how carceral mechanisms disadvantage vulnerable populations, and the need for a cultural reconceptualization of mental illness such that it is met not with criminal punishment but appropriate care.

## Background and literature review

### Mental health in prisons

Individuals with mental illness are overrepresented in incarcerated and supervised populations. Indeed, as many as 44% of those in prison have a mental health diagnosis (Cohen, 2019). Those with a serious mental illness (SMI), such as schizophrenia or severe bipolar disorder, are estimated at 15% of the incarcerated population, compared to 4–5% of the population at large (Cohen, 2019; Torrey et al., 2010; Torrey et al., 2014). In Connecticut, where this study was conducted, this number is estimated to be as much as 20.8% (Connecticut By The Numbers, 2015). A 2010 national survey examining mental illness in state correctional facilities found that in Connecticut, a person with a serious mental illness was almost twice as likely to be in jail or prison as they were to be in a hospital (Torrey et al., 2010).

This has not always been the case. The second half of the twentieth century saw a major trend in popular and political opinion away from structured, institutional care for individuals with SMI (Lamb & Weinberger, 2005). States tightened restrictions on the involuntary commitment of individuals with SMI and closed the majority of their state hospitals, the mainstay for providing long-term psychiatric care. In the absence of sufficient community services to meet the burgeoning need for intensive outpatient psychiatric treatment, many of these individuals went on to experience mental health crises in the community, which often ended in police encounters, arrests, and incarceration (Lamb & Weinberger, 2005). Furthermore, as deinstitutionalization drove down the state hospital population, the United States entered a period of mass incarceration that saw the disproportionate imprisonment of Black Americans, particularly for offenses related to substance use disorder, which impacts those with SMI at especially high rates (Alexander, 2010). Over time, due to these intersecting trends, many of those who would once have been residents of state hospitals instead came to be confined in the prison system, resulting in the staggering prevalence rates of SMI in correctional facilities seen today (Lamb & Weinberger, 2005). In the context of an overgrown carceral system and a weak social safety net, correctional facilities have become the de-facto “bottom-line” providers of mental health services, a function for which they are neither designed nor well-equipped.

The growth of correctional facilities as mental health service providers has developed alongside an unsettling ideological shift, which has come to be known as the “criminalization of mental illness.” Fisher et al. (2006) define ‘criminalization’ as “a process by which behaviors once considered legal become illegal, rendering their practitioners subject to criminal sanctions for which they were previously not at risk.” As society adopted a criminal legal response to non-normative behavior from individuals with mental illness, the behavior itself came to be conceptualized as criminal too. Fisher et al. (2006) term this a “‘re-labeling’ phenomenon, by which certain forms of deviant behavior came to be defined within a legal, rather than a psychiatric framework,” and by which “agents of social control—police and judges, would impose a criminal, rather than psychiatric definition,” on individual behavior that falls outside social norms.

Today, decades after the emergence of these trends, the prison system has become the largest provider of mental health services in the country (Reingle Gonzalez & Connell, 2014). As a result, there has been increasing attention paid to questions of what it means to treat psychiatric disorders within the carceral state and whether certain types of behavior ought to be recognized as manifestations of mental illness or instead as criminal actions requiring punishment.

As the carceral state grows to assume control over the healthcare of millions, discrepancies between its core functions and its capacity for caretaking have become evident. Despite the large proportion of mental healthcare that takes place in prisons, care remains widely varied and often substandard. One survey of state and federal prisons found that only approximately half of individuals receiving medications for a mental health disorder at admission continued to receive medication while incarcerated (Reingle Gonzalez & Connell, 2014). Another study reported that only about half of incarcerated individuals meeting criteria for “serious psychological distress” received any treatment at all, whether counseling or medication (Herman, 2019). In this way, mental health treatment suffers the effects of a poorly-equipped system and significant resource limitations, including a dearth of available clinicians, shrinking budgets, logistical challenges, and inadequate staff training surrounding mental health needs (Herman, 2019; Reingle Gonzalez & Connell, 2014).

### Mental healthcare in supervised populations

The influence of the criminal legal system on individuals’ mental healthcare also extends into the time and space beyond their release from prison. At the end of 2016, about 1 in 55 American adults – over 4.5 million people—were on parole or probation, many of whom belonged to the disproportionately large group of mentally ill individuals within the prison system (Kaeble, 2018; Skeem et al., 2006). As such, returning citizens with known mental health diagnoses are often required to engage in mental health services as a stipulation of parole or probation (Skeem et al., 2006). While this requirement is supposedly for their benefit, linking criminal legal supervision to treatment compliance conveys a complex message to individuals – that their mental illness constitutes a moral failure punishable by the criminal legal system (Skeem et al., 2003).

Furthermore, in these situations, treatment compliance may also become a prerequisite to accessing other services. Guidelines published by the U.S. Federal Courts advise the use of “positive incentives” and recommend that the parole officer acts as a “broker of services,” connecting defendants with both mental health and other community services (U.S. Courts, n.d.). In specialty probation agencies, the probation officer “actively maintains a close working relationship with treatment providers and advocates to help secure social resources (e.g. SSI, housing, transportation) for the probationer,” (Skeem et al., 2006). In this way, many potential benefits become linked to and may even be contingent upon the individual’s completion of their mandated treatment. While this may be of great benefit to individuals who may not have otherwise had access to social services, this contingency also carries moralizing undertones, implying to the individual that their access to and worthiness of public services such as SSI or supportive housing is contingent on their compliance with court-mandated mental health treatment (Skeem et al., 2003; Skeem et al., 2006).

This also functionally burdens the individual with the economic and personal costs of meeting treatment requirements. These may include paying for mandated treatment if it is not covered by the state, completing mandated medication regimens regardless of side-effects, and navigating the logistical challenges of fulfilling treatment requirements, such as transportation and paperwork, which can be further complicated by the symptoms of mental illness (U.S. Courts, n.d.). In this way, the carceral experience comes to encompass and complicate a much broader swath of people’s lives, including their housing, livelihood, and healthcare.

### Race in the U.S. prison system state

Just as individuals with mental illnesses are overrepresented in U.S. prisons and supervised populations, so are people of color– to an even greater degree. Native people are incarcerated at twice the rate of White Americans and have the highest rate of killings by police of any U.S. racial group (Schenwar & Law, 2020; The Guardian, 2015). Latinx individuals are incarcerated at 1.4 times the rate of White individuals (Nellis, 2016). Black Americans are five times more likely to be imprisoned than White Americans, and despite comprising only about 13% of the nation’s population, Black individuals make up 38.5% of those incarcerated by the federal government (Federal Bureau of Prisons, 2021; Nellis, 2016; United States Census Bureau, 2021). In the face of such glaring disproportionality, any discussion of the carceral state—including the experience of mental healthcare—will disproportionately affect people of color, particularly Black people, given their overrepresentation within the prison system.

It is also worth examining how patterns of criminalizing and burdening individuals with mental illness diagnoses may disproportionately affect people of color even prior to involvement with the criminal legal system. For instance, a study done in the Los Angeles County jail system – the country’s largest mental health provider – found that Black individuals were significantly overrepresented in the prison’s mental health treatment system relative to their representation in the general prison population (Appel et al., 2020). Such data suggest that Black Americans are not only more likely than White Americans to be incarcerated; they may also be more likely to be incarcerated with – and perhaps *for –* symptoms of mental illness. A myriad of structural factors in both public health and the prison system are likely at play in creating this pattern, including implicit racial biases of arresting officers, over-policing of Black neighborhoods, the mental health effects of structural racism, and race-based inequities in the quality of mental healthcare access and delivery. In this way, the disproportionate burden of carceral mental healthcare on Black Americans may both inform and reinforce preexisting race-based inequities in both health and incarceration.

### Conflicting goals

To be sure, prisons have developed increasingly targeted approaches to mental illness in the last several decades, incorporating on-site services and clinicians, building psychiatric blocks, and even establishing entire institutions dedicated to incarcerating and treating individuals with mental illness (Connecticut Department of Correction, n.d.-a; Connecticut Department of Correction, 2015; Herman, 2019; Lamb & Weinberger, 2005). Additionally, many institutions have implemented crisis intervention trainings for police or corrections officers to teach appropriate de-escalation during encounters with individuals experiencing mania or psychosis (Connecticut Department of Correction, 2015; Herman, 2019). Recent years have even seen the rise of mental health courts, which mandate a treatment plan rather than a criminal sentence for individuals who have broken the law in the course of an acute mental health episode (Lamb & Weinberger, 2005). However, as the carceral state grows to encompass more aspects of mental healthcare, care for both incarcerated and supervised individuals remains inconsistent, frequently insufficient, and heavily contingent on the institutional and personal styles of correctional facilities and staff (Herman, 2019; Reingle Gonzalez & Connell, 2014; Skeem et al., 2006). In Connecticut, this growth is evident in the expanding purview of carceral mental healthcare, from the 50% increase in the percentage of state-incarcerated individuals placed in a mental health prison over the past 5 years (Connecticut Department of Correction, 2021), to the growing incorporation of mental health services within carceral supervision for those on parole (Connecticut Department of Correction, 2015). Furthermore, even as carceral institutions expand the volume and breadth of their mental health services, a more pervasive and intractable problem emerges when integrating mental healthcare within the criminal legal system. On a fundamental level, the conflicting nature and goals of incarceration and mental health treatment inhibit the delivery of effective mental health services in prison or through the criminal legal system.

### Goals of mental healthcare

Though the goals of mental health treatment are varied and depend heavily on the treatment modality, institution, patient, and provider, some common best practices include: mutual goal-setting between the patient and provider, collaborative problem-solving, and an individualized, patient-centered approach (American Mental Wellness Association (AMWA), n.d..; Corey, 2016). In Connecticut, state health services are administered by the Connecticut Department of Mental Health and Addiction Services (DMHAS), whose mission statement proclaims that it aims “to promote the overall health and wellness of persons with behavioral health needs through an integrated network of holistic, comprehensive, effective, and efficient services and supports that foster *dignity, respect, and self-sufficiency* [emphasis added] in those we serve,” (Connecticut Department of Mental Health and Addiction Services, n.d.). Additionally, their statement of vision includes:

These services and supports will be *culturally responsive, attentive to trauma, built on personal, family, and community strengths, and focus on promoting persons’ recovery and wellness* [emphasis added]. Through a focus on cultivating inclusive social contexts in which individuals’ contributions will be valued, the DMHAS system will also foster a sense of *full citizenship* [emphasis added] among persons with behavioral health needs… As a result, each person will have maximal opportunities for establishing, or reestablishing, a *safe, dignified, and meaningful life in the communities of their choice* [emphasis added]. (Connecticut Department of Mental Health and Addiction Services, n.d.)

In this way, the state’s mental health language emphasizes a strengths-based, patient-centered approach that promotes healthy, strong, and empowering relationships with one’s environment, one’s community, and oneself. These goals are understandably difficult to achieve in a carceral environment aimed at punishment, rather than healing.

To be sure, these principles are ideals and as such, are rarely executed perfectly. Systems of mental healthcare face myriad challenges to effective application, even in the best of settings. The absence of carceral influence is in no way a guarantee of effective, compassionate, or patient-centered care. However, the carceral environment goes beyond merely introducing challenges to implementing these principles; it inherently contradicts them by producing an environment built on principles of control, retribution, and deterrence, which stand in fundamental contrast to therapeutic ideals of empowerment and individuality.

### Goals of criminal legal systems

The U.S. prison system performs a number of both stated and implicit functions. From a legal standpoint, the four commonly cited goals of the criminal legal system are retribution, deterrence, incapacitation, and rehabilitation (Sims, 2009). The mission statement of the Connecticut Department of Correction (n.d.-b) reflects these principles, outlining its goals as being to “protect the public, protect staff and provide safe, secure, and humane supervision of offenders with opportunities that support successful community reintegration.” In this way, prisons employ incapacitation and deterrence to accomplish the safety first of the public, then of staff, and finally of incarcerated individuals, with rehabilitation as a favorable though not necessary outcome.

In addition, researchers of carceral spaces posit that the criminal legal system also serves a number of implicit purposes, including the consolidation of political and state power, income generation, the creation and enforcement of an economic and social hierarchy that depends on a disadvantaged pool of cheap labor, and control of those perceived as being likely to pursue social change and threaten this hierarchy (Foucault, 1975; Schenwar & Law, 2020). Rachel Herzing calls this “the symbiotic relationship between public and private interests that employ imprisonment, policing, surveillance, the courts, and their attendant cultural apparatuses as a means of maintaining social, economic, and political inequities,” (Schenwar & Law, 2020, p. 8). In this way, the prison system, as “a mechanism of control and discipline,” exerts its influence in areas of society as disparate as finance, voting patterns, geographic distributions of populations, and more (Story, 2019, p. 5). In each of these domains, various actors benefit from and are invested in upholding carceral circuits and power.

These patterns also speak to the stark racial inequities seen in incarceration, including incarceration of individuals with mental illness. In *The New Jim Crow,* Michelle Alexander paints a timeline of the carceral history of the United States, describing how legislative policy and policing tactics have been both created and implemented in order to maintain racial hierarchies and suppress Black Americans. She explains that the concept of mass incarceration applies “not only to the criminal justice system but also to the large web of laws, rules, policies, and customs that control those labeled criminals both in and out of prison. Once released, former prisoners enter a hidden underworld of legalized discrimination and permanent social exclusion,” (p. 13). Brett Story (2019) describes this pattern as “racial capitalism,” or the use of racist thought, bias, and hierarchies to maintain and even naturalize the inequality that capitalism requires to function. Thus, the “twenty-first-century prison functions institutionally not only to manage but also to produce systemic unemployability across a criminalized class of African American men in particular,” (Story, 2019, p. 15). In this way, the carceral state does not simply exist to create socioeconomic barriers for whomever it happens to ensnare; rather, it functions in a very specific way to suppress and maintain a racially-delineated economic and social underclass. For all of these reasons, the criminal legal system can be imagined as existing not only for the punishment and rehabilitation of the individual, but also to protect and uphold certain structures of power and influence within various spheres of society.

### Therapeutic means to carceral ends

In the face of high demand for mental healthcare within prisons, correctional institutions have increasingly sought to integrate criminal legal and healthcare functions. For instance, the mission statement of Garner correctional institution, a mental health prison in Connecticut, states that “the staff at the facility, both custody and mental health, operates through an integrated team approach which insures a continuity of custody, care, treatment and control,” (Connecticut Department of Correction, n.d.-a). In this way, the mental health goals of care and treatment become intertwined with correctional goals of custody and control.

However, in many cases, it may not be feasible to simultaneously promote punishment and control alongside healing and growth in one individual. Incarceration removes an individual from known structures, relationships, and supports and places them in an environment poorly equipped to meet their mental health needs. Practices like solitary confinement that serve carceral functions of control and incapacitation – and that are in fact shown to be levied more often against individuals with mental illness – exacerbate symptoms of mental illness, obstruct an individual’s path to healing, and can even cause new-onset psychiatric symptoms (Grassian, 2006; Herman, 2019; Torrey et al., 2014). For individuals under community supervision, carceral monitoring and a criminal record limit one’s citizenship and self-determination – crucial aspects of mental healing – through restrictions on one’s movement, housing opportunities, economic viability, voting and jury rights, and social service eligibility (Gottschalk, 2009). The power dynamic that is inherent in the carceral system and reinforced through rigorous rules, limitations on autonomy, and constant supervision situates the individual in a position of powerlessness and creates a hostile environment that is inconducive to healing. As Lamb and Weinberger (2005) aptly state, “jails and prisons have been established to mete out punishment and to protect society; the corrections milieu is limited in its ability to be therapeutic.”

The tension between carceral and mental health treatment goals grows even starker when understood in the context of the implicit goals of the carceral state, such as economic stratification and the “construction of disposable people in our social worlds,” (Story, 2019, p. 11). When considering the role of mass incarceration in producing and maintaining racial capitalism, unemployability, and the creation of a social and economic underclass, it becomes evident that those with mental illnesses – particularly people of color – who already face significant discrimination and accessibility challenges, are particularly targeted by these carceral goals, which operate in direct conflict with therapeutic goals of empowerment and self-sufficiency.

Ultimately, when mental health treatment is incorporated within and made an agent of the prison system, mental health goals become secondary to those of that system or are abandoned entirely. Bringing mental healthcare within the carceral state, an institution oriented toward control and retribution, fundamentally conflicts with the therapeutic ideals of patient empowerment and agency and ontologically transforms the nature and purpose of that care. In the words of Maya Schenwar and Victoria Law in *A Prison by Any Other Name,* this illustrates “how pervasive incarceration has become that even many of the alternatives, which are couched in the language of healing, actually rely on forcible confinement, surveillance, and utter control,” (Schenwar & Law, 2020, p. 18).

In this paper, we build on this understanding of the fundamental incompatibility of the goals of the carceral state with those of mental health treatment. We draw on qualitative interviews with nine individuals who illuminate this divide. Their perspectives and experiences illustrate the theoretical concept that mental healthcare deployed via the criminal legal system operates as a mechanism of carceral power that reinforces existing hierarchies. We first examine the ways in which participant testimonies illustrate how their care reflects carceral goals of retribution over healing and control over empowerment. We also explore the functional deficiencies of such mental healthcare through patterns of diagnostic ambiguity and ill-fitting treatment described in participant accounts, as well as de novo experiences of mental illness brought on by incarceration. Additionally, we contextualize these experiences within broader patterns of racial hierarchies that exist within and are reinforced by carceral systems. Finally, we highlight the ways that participants respond to, optimize, and derive benefit from non-ideal treatment.

## Methods

### Research setting and design

The parent study for this analysis was the Justice, Housing, and Health Study (JustHouHS), a multi-institutional, mixed methods, longitudinal study to examine the intersecting impacts of mass incarceration and housing vulnerability on health outcomes among low income residents of New Haven, Connecticut who are 18 years of age or older. Participants (*n* = 400) were recruited via referral from service providers, flyers placed in public, and snowball sampling. Given the study’s focus, the sample was stratified to include 200 individuals who reported recent release from prison (within 1 year of the date of screening).

### Data collection

The study consisted of a quantitative arm, in which participants took a self-administered survey covering various topics, including physical and mental health, income and economic factors, housing, interactions with and opinions on the criminal legal system, social and community engagement, family history, sexual relationships, substance use, HIV/AIDS awareness and risk factors, trauma, and spiritual beliefs. Participants returned at six-month intervals to take the survey over the course of two and a half years. Additionally, a subset of 54 participants who indicated interest were purposively selected to participate in the qualitative arm, which involved one-on-one, in-depth interviews of about one hour’s length on similar topics covered in the survey. These interviews took place in New Haven and were conducted by two members of the research team (PS and AR). These interviews were also conducted at six-month intervals for four iterations. Recorded interviews were transcribed by a contracted transcription service and checked for accuracy by 1–2 members of the team.

### Data analysis

Following transcription, interviews were uploaded into Nvivo 12 (2018), a data management software platform. They were then coded by team members according to 19 index codes developed from the original study questions.

The present analysis is based on findings from the qualitative arm of the study. It focuses on interviews with nine participants. This group of nine included the subset of participants in the interview cohort who both self-reported a psychiatric diagnosis at some point in their life and reflected on the intersection of this diagnosis with their criminal legal experiences in their interviews. Of these nine participants, two identify as female, and seven identify as male. In terms of race and ethnicity, three participants identify as African-American, one identifies as White, and five identify as multiracial/other, four of whom also identify as Hispanic. The current analysis includes 34 interviews with these nine participants over the course of two years, from November 2017 to November 2019. The longitudinal nature of the study allowed not only for discussions of changes in life circumstances, but also for further exploration of previously mentioned events and topics as the interviewer/participant relationship deepened (Barrington et al., 2021). Through a process of open-coding, memo-writing, and analytical meetings about specific transcripts, thematic sub-codes specific to issues of mental health were developed and applied to the interview transcripts of the nine participants to further stratify the data. These sub-codes were used to identify participant quotes of particular relevance to the specific topics included in this analysis. Examples of sub-codes include: mental healthcare in prison, mandated mental healthcare, medication, ill-fitting treatment, and trauma. The following results were derived from participant testimonies and opinions that were identified through this process.

## Results

### Ontological effects of incorporating mental healthcare within the carceral state

The stated goal of mental healthcare—to assist the client in reaching healing and empowerment—and the goals of the carceral system—to control and correct—are fundamentally at odds. Bringing mental health treatment under the carceral purview renders this treatment a mechanism of growing and maintaining the carceral state and of enacting its goals of control and punishment. Our analysis of participant perceptions of their mental health care while in a supervised population clearly expresses these themes.

#### Punitive rather than therapeutic

When implemented by the carceral state, practices meant to be therapeutic in other contexts are experienced as punitive by both the representatives of the carceral state and those it incarcerates. This is clearly conveyed by Leah, a 25-year-old woman, who described an instance during her incarceration when she was committed to the psychiatric ward and spent a period of time in solitary confinement following an altercation with a corrections officer. In recounting this experience, she highlighted her heightened sense of confinement while in the psychiatric ward:

From Seg, I went to the turtle tank, which is where they don't give you no clothes. You've got like this big robe with Velcro, and they sit you in there for like a day. And then from there I went to the Psych ward for like 15 days. They're legally only supposed to hold you there seven, and I was there 15 days. The only reason why I got out was because I slid a note under the door to [corrections officer] and I had told him that I know I'm being held here against my will, and now it’s a matter of legality, and if I need to, I will have my niece, who's in the military. That same day I got put out, and I got put on the compound…

The way Leah and the corrections officer communicated about her confinement in the psychiatric ward suggests that both of them understood this to be a disciplinary measure in response to her behavior, rather than a genuinely therapeutic measure undertaken to facilitate her healing and rehabilitation.

Similarly, Carter, a 33-year-old man, described being transferred to a maximum-security prison after displaying symptoms of a schizophrenia exacerbation. Despite having a known diagnosis, he was not receiving medication in prison and began experiencing symptoms of his condition. As punishment for his behavior, he was transferred to a higher-security facility. Although he was later able to successfully petition the prison to resume his medical therapy, he still remained in a high-security facility even after his mental health condition was recognized and treated. He stated:

I was a level three in a level four or five prison…Like, I had like 10 months left but they had me in jail with people that was doing 65 and 100 years, like people that got homicides and everything like that. You got – I got 10 months. You got me around people that's doing 35 and all of this.

In this way, Carter described a sense of being overzealously punished and classified as a higher-order criminal as a result of the manifestations of his mental illness. While mental illness may often go undiagnosed in prison, Carter’s case illustrates that even when mental health conditions are recognized, they may still be handled by the carceral system in a punitive manner, reinforcing both the systemic criminalization of mental illness and the dominance of carceral goals like retribution over therapeutic goals for supervised individuals.

These patterns were also identified in community settings by individuals on parole or probation. Cora, a 36-year-old woman, described requesting a signature from a mandated mental health provider to confirm her treatment adherence. Despite her compliance with the program, her request was met with scorn and hostility. She recalled,

Her demeanor and her whole approach to me was so disrespectful … I'm coming here respectful and coming on time and coming dressed appropriate. I'm not coming flamboyant, I'm not coming high. I do my urines clean. I'm doing my end of the program so if you're supposed to be in my corner, supposed to [be] a social worker, you're supposed to be, like, meet these goals and this and that; you're tell me that on one end but you're not even helping me accomplish one goal with just a signature so you're not really, like, on the same page with what you say. So you're not practicing what you're preaching to me. So I feel like how am I gonna listen to a place like this, like what kind of program is this really?

The adversarial and derisive behavior of Cora’s provider stood in stark contrast with Cora’s expectations of a mental health provider: to help her set and accomplish goals and to be “in my corner.” In this way, Cora’s provider – rather than allying with Cora to provide therapeutic care –perpetuated the punitive tone and the message of moral inferiority that is characteristic of carceral systems.

#### Control rather than empowerment

Many participant stories also reflected perceptions that their mental health treatment was part of a broader effort to control them, rather than a means of empowering them to achieve mental health and personal healing.

Lee, a 56-year-old man, discussed an encounter in which he attempted to refuse a psychiatric evaluation after being involved in an altercation with another incarcerated individual. In response to his attempted refusal, the Correctional Emergency Response Team (CERT) was brought in to force him to comply, which resulted in Lee being pepper sprayed, beaten, stripped, and put into a four-point restraint. He recalled,

After they took the restraints off and all that and then they said, ‘The psychiatrist want to see you.’ I’m already down there now. Don’t you know, when I got out and went in there to see the psychiatrist, don’t you know he asked me, he said, “How can I help you?” [Laughter] I could have killed him. I just got assaulted and beat up and I didn’t ask to see you in the first place, and he’s gonna talk about, “How can I help you?”

On the surface, the therapist’s question suggests both a desire to help Lee and a pretense that Lee has some degree of agency regarding how he would like to be helped. However, the act of violently forcing Lee to participate in this “therapeutic” interaction undercuts the theoretical empowerment offered by mental health treatment in this carceral space and reinforces the controlling nature of the interaction.

The carceral state’s use of mental health treatment to control was also apparent in individuals’ accounts of treatment while on parole and probation. For instance, Logan, a 35-year-old male, described feeling as though the mental health of supervised populations was being sabotaged to reinforce carceral power. While on probation, he experienced a conflict with his roommates, in which his psychiatrist and case workers became involved. In describing the ways that case managers handle the personal issues of individuals on probation, Logan stated,

Logan: It's just screwed up, on top of screwed up, on top of screwed up, on top of screwed up. But we get paid money. As long as things are screwed up, we get paid. So we want to kind of keep them screwed up. You know what I mean?

Interviewer: It gives them a job?

Logan: Yeah. And if the—you know, they—they kind of betting that the situation would get worse. You know what I mean? By that I mean, I'm either going back to jail or I'm going to be hospitalized.

Interviewer: That's what they assume?

Logan: That's what they're—that's how they're playing it. You know what I mean?

Interviewer: Instead of, like, you're doing better.

Logan: Yeah.

From Logan’s perspective, individuals who are presumably employed to work in concert with mental health providers to support the wellbeing of individuals on probation actually benefit from setbacks in the mental health and social wellbeing of their clients. In this way, mental health treatment is rendered unproductive when contained within a system designed to protect and reinforce its own economic gains and those of individuals who are invested in the system.

This theme of ‘control rather than empowerment’ also arose frequently in discussions of medication. Carter described how his medication regimen was modified in prison, stating, “they really went up on my meds when I went to jail. I didn’t feel the same.” He elaborated on this, saying,

Yeah, ‘cause when you went to jail they were like we're gonna try you on this and I feel like they using me as a lab rat… Risperdal had me zombied out. I didn't like that feeling. I was quiet, wouldn't talk, moving slow…I think they overmedicate you in there so they won't have to deal with you.

Logan voiced a similar perspective when discussing how caseworkers manage mentally ill individuals mandated to treatment: “What can they do? You know? When you got guys that you, like, you know, medicate them and just hope they shut up. You know? Be quiet.” Testimonies like these illuminate how pharmacological treatment, like other modalities of mental healthcare, can be made into a tool of the prison system to suppress and control individuals and consolidate its own power.

Mental healthcare delivered via the carceral state may appear to offer the same components as community-based treatment. However, the experiences described by these individuals emphasize the ways in which it lacks the core therapeutic orientation to help the client achieve healing and empowerment, instead existing to reinforce the carceral goals of punishment and control.

### Effects on service delivery

When mental healthcare is engaged without a patient-centered focus, carceral goals of discipline and control supersede therapeutic goals of healing and empowerment. Beyond the potential harm of receiving care that is not primarily meant to benefit the patient, this shift may also result in ineffective service delivery, including ambiguous diagnoses or ill-fitting treatment.

#### Diagnostic ambiguity

Within the carceral setting, the mental health diagnostic process exists along a continuum of ostensibly therapeutic activities that are implemented toward carceral ends. Thus, rather than serving the goal of better understanding and treating the individual, the diagnostic process becomes a means of facilitating control over individuals, namely by labeling and ostracizing them. As such, diagnostic confusion was a prominent pattern in participant narratives, with many reporting that they had received one or more diagnoses they felt were inappropriate or inconsistent. Carter’s experience provides one example. He reported receiving a psychiatric evaluation while shackled to a hospital bed and under the influence of PCP:

I just told 'em like this, “You diagnosed me when I was under the influence. That gives you symptoms like that, so I don't know why you're wanting to evaluate me. Don't evaluate me when I'm blacked out, having symptoms, high. You had me cuffed to a bed, so I was fine. You should've just let me come down, sit for a little while, and then evaluate me. Don't just put me on meds and then threaten me, you're gonna lock me up and do all this or send me back to jail if I don't take meds. That's crazy, but at least evaluate me when I'm like this. Don't evaluate me when I'm under the influence. That gives you them symptoms.”

In his case, his hasty diagnosis enabled the criminal legal system to apply a mandated treatment plan as a form of control and to impose punishments in response to nonadherence. Carter goes on to explain, “I violated probation and all of that so many times for not taking medication.” Clearly the experience initiated at the hospital was not unique but part of a broader pattern of being punished for medication nonadherence. The mandating of medication and the threat of incarceration for noncompliance are both leverage by which this inappropriately-applied diagnosis secures and reinforces carceral control. The diagnosis did not make sense to Carter, but it did not need to do so to accomplish the goals of the carceral system.

Similarly, Logan reported that at various times throughout his life, he received diagnoses that he questioned or outright rejected. However, his entry into the criminal legal system later in life allowed him to see that his legal involvement “adds something else to it.” He indicated that the diagnoses applied to him in the setting of his criminal legal interactions served a different kind of purpose, stating, “I think the whole thing, they’re just finding excuses to kind of make you seem weird or to categorize you or to put you into certain, um, like, you know, ‘He goes in this box.’ ‘He fits this category.’” He also expressed that he felt that having his diagnosis applied within a criminal legal context resulted in the conclusion that “He must be dangerous.” Logan perceived that the true goal of this diagnosis was not to better understand, normalize, and support his condition, but rather to assign labels that rendered his behaviors simultaneously strange, threatening, and manageable. In this way, the use of his diagnosis within carceral healthcare reinforces the recipient as both needing to be and able to be controlled.

Similarly, Cora described a pattern of being repeatedly labeled with a diagnosis of schizophrenia while incarcerated, even though she understood her symptoms to be due to substance use. She felt she was wrongfully placed in the mental health ward at the onset of her incarcerations yet still did not have access to proper treatment. She, like Carter, described being diagnosed in the setting of substance use, “going in and out of the mental hospital at the time I was using” and stated,

They say I got schizophrenia and psychosis but they always say it’s through chemical dependency throughout that, so I really wouldn’t say that, I guess, but when I go to jail, they do say that, so they usually put me in mental health at first because they say, you know, like I'm schizophrenic and whatever, I guess like a danger – whatever. So I just…okay, fine, whatever you say, but they don't give me nothing for it either. I mean if I am psychosis, all that stuff -- I guess it looks good to them on paper but they don't give me anything for it anyway so I don't know what that's supposed to mean.

In this way, Cora, much like Logan, perceived the prison administration to be using her diagnosis to label her as “dangerous” and to sort her into a category that “looks good on paper” for the prison, rather than a category that will result in her getting the appropriate treatment. Additionally, she did not even receive appropriate treatment for the category she *was* sorted into, again demonstrating that these diagnoses exist for the benefit of the prison system, rather than the individual.

Another participant, Jackson, a 24-year-old male, expressed frustration with his ever-changing diagnoses. In reflecting upon the many mental health evaluations he had received as a result of childhood encounters with law enforcement, he stated, “They changed –come on, they changed the diagnosis like every single year. They changed what I was. Like some of them sounded legitimate, like ADHD and like a spectrum autism. Understandable, but they just kept changing, changing, changing.”

These accounts of diagnostic ambiguity reflect the ways in which clinicians acting on behalf of the carceral state fail to do the work of understanding the individual and their symptoms, instead applying diagnoses that best serve the needs of the carceral state in that moment, leaving individuals confused, skeptical of their care, and uncertain of whether they will receive effective treatment.

#### Ill-fitting treatment

Carceral mental healthcare, as an entity aimed at controlling and punishing individuals rather than healing and empowering them, often fails to meet their needs. This is clearly revealed in participant descriptions of inappropriate care they received while incarcerated. In Lee’s case (discussed above), his experience of being forcibly brought to the prison psychiatrist highlights the discrepancies between his needs and his prison-mediated therapy. He recalled,

The psychiatrist really got me, talking about how can he help me. He don’t know. He lucky that he had me in the cage – that cage, they put you in the cage. He talking about how can I help you. How can you help me, I didn’t even ask to see you for one thing? And he said, well you take him back. And I went right back.

For all the struggle and trauma that went into bringing Lee before a provider, he ended up being sent away almost immediately without any treatment or counseling. Because the goal of carceral mental healthcare – to control Lee’s behavior – had already been accomplished, his treatment was abandoned before it started, thus failing to meet his mental health needs.

Participant narratives also reveal how the trend of ill-fitting treatment extends to community-implemented mandated care for individuals on parole or probation. In Jackson’s case, he found his mandated group therapy to be not just ineffective but also directly counter-productive:

It was just like how can you, you know, try to help me out but put me around people that are even in the same boat or doing worse? Like how is that benefiting –how is that motivating me?... It was just I can't be around these people and I can't be talking about that 24/7. Like how can you try to better yourself and talk about drugs and this and that the whole time?

Jackson’s perception that his treatment was not well-suited to his needs and his ensuing frustration reflect how carceral control over mental healthcare results in the loss of individual autonomy.

Similarly, Leah and Ryan both expressed ambivalence about their mandated talk therapy. After hearing from a clinician that she would like to treat him for “trauma,” Ryan, a 36-year-old male, strongly rejected the idea:

No, I don’t need that. I mean, if I need it I would do it. But I don’t need talking about my – that’s in the past. I already closed that chapter right?... I don’t pay attention to that. I focus on what happens now and what I’m gonna do next and what I’m gonna do to prevent that to happen again.

Likewise, Leah discussed the reasons for her aversion to talk therapy:

I don't like talking to people… It's hard. Like, I really like acting like it did not happen. [Laughs] I really do. Like when I lived in –I swear to goodness I was happy in California, I was happy in Florida, and I was happy in New Mexico. Why? Because I could be a completely different person.

While exploring trauma with a trusted provider can be a powerful source of healing, a patient must engage this process willingly when they are emotionally ready and equipped to do so and when they have access to a provider they trust. In both cases, these participants expressed that being made to relive past events with a mandated provider was not what they needed at the time and in fact disrupted their ability to cope or function in their current situation.

Furthermore, Cora, who also received mandated treatment while on probation, described a feeling of being “in the hands” of her providers and a pattern of going through the motions of mandated care without deriving any benefit.

I would say the providers that are mandated provided to me, like if I go to probation and they're like show up to this, please, do this, do that, the court says and they stipulate mental follow-ups, which are mental evaluations, and whoever they send me to and whatever – the people that I just end up in their hands and I just deal with them and, you know, comply and show up and whatever. You know, they ask me a question I'll say yea, nay, whatever, but it's not like I feel like it's doing nothing for me or I want to see them, you know, so…

She also expressed frustration with the mismatch between her mandated care and her actual needs:

I know that, you know, years from now a lot of the things legally were put in place by faults of my own, but at this point how do I get past that? Like okay, on paper I'm this person but I'm more than capable of handing you a sticker or greeting you at Walmart or-or passing you a plastic bag ‘cause you have returns today. I can have a normal conversation, I can count to 10, like there's things I can do. I see people—secretary or greeters and things and I can do above and beyond, but because of my record I get hindered and then, you know, it falls into part and everything… probation's answer is to send me to this place to do outpatient. Like how much outpatient can I do?

In Cora’s case, she felt that being mandated to treatment at all was ill-suited to her needs. While she may have derived benefit from it at one point, it has become repetitive and ineffective and may even have begun to obstruct her goal of finding work. She clearly expressed frustrations with the barriers created by her criminal record and lack of opportunities. However, the carceral system continued to direct her to redundant treatment programs she did not need rather than addressing her stated needs. In all of these cases, the carceral healthcare system denied participants the opportunity to make their own choices and to freely participate in their own care, an important component of effective treatment, and instead offered mental healthcare that functioned primarily to benefit the carceral state at the expense of the individual.

### The prison experience as trauma

The testimonies of our participants illuminate the obstructive nature of the carceral system for effective mental health service delivery. However, the harmful influence of the carceral state does not end with ineffective treatment for preexisting conditions. The trauma engendered by the carceral system can create de novo mental health problems for individuals. Thus, interactions with the prison system can constitute a setback to mental health not only through the subversion of therapeutic goals, but also by creating trauma in and of itself.

Cora’s story provides a compelling example. She shared with her interviewer a traumatic encounter with a police officer prior to her incarceration. She and her friend were stopped by an officer without reason, assaulted with Mace, and sprayed with a fire hose:

I've been beaten by the [City] police very, very severely. I had a black eye, I had taser marks, I had a pin needle mark. He stuck me with the pin on his badge and I also have pictures of that, that the [Regional] Correctional Facility took as a precaution to them so I won't sue them saying that their officers did it, ‘cause I was horrified. I had to take pictures kind of – you know, I had my top off because a lot of bruises were on my upper part body and the CO – she was crying with me. She said, "You know, I'm sorry that that happened to you but we have to so you won't, you know, take any action against us ‘cause it didn't – you came in like this, it didn't happen here.

Cora’s account of being beaten so badly that the institution incarcerating her was compelled to document proof that they were not responsible illustrates the reality and severity of the trauma that can be produced by the carceral system.

Similarly, Carter stated that prison “messed me up.” Throughout his interviews he recounted various traumatic experiences, including overhearing sexual assaults, losing his mother to cancer while incarcerated, spending 30 days in solitary confinement, and being beaten by his CO while the supervising nurse looked the other way. These experiences have had lasting repercussions; he stated, “I always have nightmares about getting killed.” In this way, the lingering trauma of the prison experience has stayed with Carter and constitutes a psychological hurdle he did not face before.

Leah similarly expressed that the prison experience can be traumatizing. She described being physically forced into a dirty shower by a corrections officer, as well as another traumatic incident that remained off-record due to its sensitivity. In reflecting on these experiences, she stated, “Being in jail can be traumatic though for people. It can be – it’s like a shell shock, it’s like, boom. And if you don’t adapt well, it’s like, oh no. No, no, no, no.”

Finally, Lee’s account of being forced by the CERT team to see the prison psychiatrist speaks to a traumatic experience of physical abuse and helplessness. As previously described, he was tackled, beaten, handcuffed, stripped naked, and choked, all in the course of this incident.

So, I hear them out there, there they go one, there was two, three, and then snatched open the door and ran in there and jumped on my back and started hitting me in my face, put handcuffs on me, punching me all on my side, put my knee in my back, punching me…my face was swollen – IPM – that’s what they call it. Something like that… I already had a jumper on, they drag me all the way backwards all the way to there and they put me in the four-point restraints. I didn’t even have no hands. They had me on my back and they put me in the four-point restraint. I ain’t even have no circulation in my hand. … I didn’t have no feeling in my hand for a good while. So, I’m laying there. So, the other one had his elbow on my throat. You know, trying to cut my air circulation off while they was cutting the jumper off. They was cutting it off, ripping it off…

Much like Cora, Leah, and Carter, Lee recounts an experience of trauma as a result of interaction with the prison system. However, Lee’s experience goes even further, to demonstrate that the trauma enacted by the prison system not only *offsets* the benefit of mental health services in prison, but that this trauma can even be *produced by* these mental health services. The psychiatric treatment supposedly offered for Lee’s benefit was the very encounter that created trauma. These testimonies thus illustrate how the prison system undermines mental health not only by transforming healing modalities, but also by creating new trauma that poses an injury to individuals’ mental health.

### Race and carceral mental healthcare

As noted earlier, people of color – particularly Black Americans – are disproportionately targeted and confined by the criminal legal system. Thus, in addition to the many other burdens of disproportionate incarceration, the Black population also bears the brunt of the myriad problems that arise from carceral mental healthcare. The experiences of Isaiah and Sammy regarding their interactions with police around mental health speak to how the intersection of mental healthcare and the carceral state can disadvantage people of color.

In his interview, Isaiah, a 46-year-old Black man, described experiencing symptoms of mania in the context of a known bipolar disorder diagnosis. Isaiah recognized what was happening and went to the emergency room several times to seek psychiatric care. Rather than admit him and treat his condition, however, the hospital security attempted to force him to leave, ultimately resulting in an altercation and Isaiah’s arrest. In discussing the incident, he says,

I don't believe that everybody's racist but I think certain people in certain positions... can be, you understand, and it's disheartening. And they use their position to do whatever that they feel... Because if I was going to the hospital three or four times there was no reason why they shouldn't have admitted me, you understand? I was reaching out for help and they did not help me.

Despite persistently seeking necessary healthcare as would seem appropriate, Isaiah’s symptoms and persistence were perceived as criminal, and he was instead diverted to the criminal legal system. He attributes this response to the racism of the hospital staff and further acknowledges how they use their power in the health care system to exercise their racism – to “do whatever they feel.” This reflection underscores the fact that Isaiah did not merely experience anti-Black discrimination on its own; rather, it occurred in the context of a separate power imbalance: the hierarchy between a patient and medical provider. The medical power imbalance, together with the involvement of the criminal legal system, served to magnify the harm caused to Isaiah by the racial bias of individual providers.

Sammy, a 60-year-old White man, had very different experiences with the carceral system. Despite having recurrent struggles with substance use, including a few police encounters, he was never incarcerated. Reflecting on this, he stated,

Well, I mean just in my own case I know, like, being caught maybe once or twice buying drugs on the street, I mean I think because I was White it was different. It's just like, you know, get some help, go to a meeting or something like that. That's the way they treated with me.

In this way, Sammy perceived the police and the carceral system at large as having reacted to his substance use with a race-based script. His story is almost an inverse of Isaiah’s. Isaiah presented to a healthcare setting to seek help for a mental illness but was instead diverted to the prison system. By contrast, Sammy’s encounters with the criminal legal system took place in a non-healthcare setting and could easily have resulted in his incarceration. However, because he was White, he was diverted from criminal legal involvement, and his substance use was treated as a health issue, rather than a crime. In other words, as a White man, he was able to access mental healthcare outside of the carceral space. Furthermore, he continued to benefit from extensive life-saving treatment and services related to his diagnoses throughout his life without ever being subjected to the same criminalization that Isaiah experienced.

The contrast between their stories illustrates how the criminalization of mental illness does not apply to all people in the same way; rather, its application reflects both individual and systemic racism. As Fisher et al. (2006) note, “how the ‘problem’ of offenders with mental illness is framed plays a major role in the interventions proposed to address it.” In this way, a race-based script for the framing of atypical behavior can result in life-changing differences in criminal legal involvement for individuals of different races, such as Isaiah and Sammy.

### Outcomes / strengths-based analysis: adapting and using Carceral mental healthcare to individual advantage

Despite the myriad ways in which carceral mental healthcare serves to disempower and control individuals, participants found just as many ways to exercise agency and resistance in these situations. Whether by taking ownership of their own care as possible, engaging mandated care on their own terms, or using mandated mental health treatment to access linked social services, participants found ways to identify and fulfill their own needs, rather than rejecting mental healthcare as a whole. The following sections expand upon each of these examples in greater detail.

#### Taking ownership of one’s own mental healthcare after criminal legal involvement

Many participants described adapting their formerly problematic, prison-oriented care to serve their own needs, goals, and wellbeing. For example, Isaiah, who was arrested while seeking mental healthcare at a hospital, was able to get established on a medication regimen that effectively controlled his symptoms while in prison, after a long process of advocating for himself. Furthermore, he has continued investing in and personalizing his mental healthcare after gaining freedom from the criminal legal system. He stated of his current clinician, “I love it. I enjoy going to see her.” Regarding his medication, he shared, “I take it on my own behalf. I feel like that’s part of my freedom…” While his mental healthcare was once incorporated within his prison experience, Isaiah now freely engages with his care outside the criminal legal system and of his own volition and thus associates it with freedom.

Similarly, Carter described a process of making his mental healthcare his own once he was no longer under the restrictions of parole. Despite his frustrations with mandated treatment and his multiple parole violations for refusing medications that made him feel like a “zombie,” Carter did not reject mental healthcare when his parole ended. Rather, he engaged his clinicians in a discussion about a more appropriate medication and continued his involvement in therapy. He describes therapy’s main benefit as having “just somebody to talk to. Sometimes you need people to run stuff by before you do something crazy. You need somebody to talk to.”

For both Isaiah and Carter, the criminal legal system provided a doorway to mental healthcare, however imperfect or imperfectly motivated, which they were then able to continue on their own terms and to their own benefit.

#### Ambivalent or piecemeal engagement in mandated services

Unlike Carter and Isaiah, individuals who remain under parole or probation may not have as much flexibility to take control of their mental healthcare. Still, while mandated treatment was often ill-suited to participants’ needs, many endorsed a degree of willingness to derive some benefit from it, even while recognizing that the treatment and the system administering it were flawed.

For instance, despite his deep distrust of his caseworkers and their motives, Logan affirmed that his participation in his mental healthcare was at least partially voluntary. He stated, “if I felt it didn’t help me … it wouldn’t matter to me, like, going to jail for a year or two,” and, “I just take from it what I can.” Logan judged that being reincarcerated for noncompliance would be favorable to participating in aimless treatment, so his continued participation in care indicates that he derives some degree of benefit from the care, even if it is complicated by the problems he discussed.

Ryan described a similar sentiment toward his mandated therapy: “I mean, I can learn from them or I just grab what I need and the rest, I throw it out.” Despite an incomplete acceptance of the principles and premise of his therapy, he acknowledged there may still be something to learn. While distrusting of carceral agents and resistant to mandated care, both Logan and Ryan chose to selectively engage their care, determining for themselves what was most valuable for their needs.

#### Acquiring social services distinct from mental healthcare via mandated treatment

Finally, a number of participants described using their mandated mental healthcare to gain access to other services or to achieve goals distinct from mental health. One prominent example was access to social services. For example, despite Leah’s reluctance to participate in therapy, her therapist did help her apply for State Administered General Assistance (SAGA) for her family. Similarly, Carter used his contact with his case manager as a means of maintaining housing security, stating that this allowed him to be diverted to Crisis and Respite in the event of a schizophrenic episode, rather than going to jail and potentially losing his home.

I'm gonna keep my case manager longer when I move out and see how things go, ‘cause last time when I had my own apartment before I went back to jail if I get too worked up from being a paranoid schizophrenic, too nervous, anything, they let me go to Crisis & Respite and stay for two weeks…And then they let me come back, but you gotta keep your case manager. They give you the choice if you want to keep the case manager or not. I keep it ‘cause if something goes wrong at least you've got something to fall back on.

Cora described using her mandated inpatient treatment program to connect with a case worker, who supported her in various ways, providing financial advice and rides and helping her to find housing and employment.

Really she helps me with anything that I want, like if I want to get back into school. We did a list of short-term goals and long-term goals. She's trying to help me get my license. They don't do it personally, they don't fund it personally, but we're gonna find the avenue that we have to go to and get it done. And she brings me for rides like to appointments really. It's really…Yeah. And she doesn't leave my side until I find either work or housing. That's what she told me.

Interviewer: And has this – have you ever been assigned someone like that before?

Cora: Never. I feel like if this would've happened years ago, it would’ve helped me, you know, have another advocate.

In these ways, individuals exercised some control of mental health treatment avenues to access much-needed social and personal resources, even outside the realm of mental health.

Overall, participants exhibited a wide variety of responses and adaptations to their mental healthcare, reflecting the diversity of the needs and experiences of formerly incarcerated individuals. While each participant’s story is different, these responses often represented the result of years of repeatedly encountering and iteratively adapting to carceral mental healthcare.

## Discussion

These descriptions of how carceral authority extends into spaces of mental healthcare are reminiscent of the patterns described by Brett Story, who conceptualizes carceral space as “a set of relationships dispersed across a set of landscapes we don’t always view or conceive of as carceral,” (Story, 2019, p. 6). Through controlling individuals’ access to and experience of mental healthcare, the carceral state transforms health and community environments into carceral spaces and serves to accomplish “the relocation of carceral authority from spaces of detention to the churches, homes, schools and local nonprofits currently tasked with everything from reentry programming to the surveillance of movement and behavior,” (Story, 2019, p. 163). In this way, the incorporation of mental healthcare and related services within the purview of the carceral state enables a greater degree of state control over individuals and their communities, both during incarceration and after their release from prison.

In examining these trends, it is worth interrogating the functionality of incorporating mental healthcare within the prison system as a means of punishment and control. In other words, what unique function does carcerally-adapted mental healthcare serve for the prison system? The fervor with which carceral agents search for and manage psychiatric problems for incarcerated individuals suggests one theory. In his analysis of the creation of psychiatric diagnoses in prison, Joseph Galanek (2013) describes how traditional diagnostic processes may not be appropriate for incarcerated populations. While the DSM’s original model of mental pathology assumes a “middle-class Anglo prototype,” Galanek argues that its diagnostic limitations are exposed when one attempts to apply it to populations from vastly different backgrounds. He suggests that the intersection of various personal, developmental, environmental, and structural factors around and within incarcerated individuals, many of whom have suffered poverty, racism, abuse, trauma, or been the victims of crimes themselves, “creates a substantially different prototypical case than those presumed in the DSM’s categories,” rendering a purely neuropsychiatric explanation of criminalized behavior overly reductive (Galanek, 2013).

This idea is reflected in the three participant accounts of diagnostic ambiguity. In these accounts, there is both a perceived emphasis on arriving at a diagnosis—any diagnosis— to which their behavior could be unambiguously attributed, apart from any role that environment or social structures may have played, and an inability of the clinicians acting on behalf of the carceral state to do so. This reflects the ways in which carceral mental healthcare can overemphasize the individual factors of mental health, eschewing the importance of one’s environment, relationships, social context, and experiences in creating mental health issues. Similarly, many of the ill-fitting treatment plans described by participants relied primarily on pharmacotherapy or individual psychotherapy, rather than employing a holistic approach to address criminalized behavior patterns, which are often as much a result of structural inequality and developmental context as of individual psychiatric factors.

To be sure, overmedication and oversimplification of mental illness and treatment are by no means exclusive to carceral mental health care. However, the heightened degree of focus on establishing a psychiatric diagnosis and identifying a psychopathology-focused treatment seen in these participant accounts points to a unique function of carceral mental healthcare: to locate the source of individuals’ mental struggles within themselves and their own psychiatric ‘pathology,’ rather than acknowledging the social, economic, and relational factors that come to bear on a person’s mental health – to which the carceral state directly contributes. Social patterns of poverty, exclusion, and racism, which act as tremendous stressors to individual mental health, are also deeply intertwined with the prison system, which recapitulates these patterns by design. In this way, the carceral state may in fact have a role in creating the patterns and behaviors of mental illness that it purports to treat in incarcerated individuals. In pathologizing criminalized behavior and situating both the cause of and the solution for this behavior within the individual, the carceral system absolves itself of any blame in creating or exacerbating mental health problems, as well as any responsibility to remediate its negative societal effects.

Despite the many downfalls of mental healthcare that is mandated or implemented within the carceral state, many participants nevertheless found ways to use this system to gain certain benefits. This resourcefulness on the part of strategic individuals does not absolve the prison system of its pattern of injustice toward people with a mental illness, particularly those who are people of color. Nor does it validate the prison system as an appropriate provider of mental healthcare. Rather, it reflects an implicit understanding on the part of these individuals that mental healthcare is an entity that is intertwined with, yet distinct from, the prison system. In selectively engaging with the parts of mental healthcare that they find to be useful or helpful, individuals demonstrate their recognition – or at least hope – that the benefits of mental health can be divested from the prison system and its goals.

This analysis was constrained by several limitations. First, the small size of our sample and our narrow geographic scope were conducive to an in-depth, context-driven analysis, but these factors also inherently limit the generalizability of these results. Thus, replication of this project in different regions and populations could be a way to expand and compare results in this area of study. Additionally, while this study took place over two years, we recognize that mental illness and criminal legal involvement impact individuals across the entire life span. As a result, this analysis represents only a snapshot of this impact for these participants, many of whom were in different stages of processing these experiences. This limitation presents some opportunities for future research, namely a more prolonged observation of post-incarceration engagement with mental healthcare and the incorporation of other longitudinal factors such as recidivism, changes in service utilization, and recovery parameters.

Finally, these findings must be understood in light of the significant limitations posed by the positionality of the researchers. Despite our best efforts to remain true to the intentions, opinions, and experiences of the study participants, this analysis was conducted by individuals other than the participants themselves and thus is subject to the inevitable influence of our own worldviews and assumptions. For this reason, this analysis is limited by the lack of those with lived experience as research team members and analysts. Moreover, we struggle with the tension between protecting the anonymity of study participants and giving credit for and full ownership of this paper to the individuals to whom these stories truly belong. While these limitations necessitate the positioning of ourselves as “authors,” we recognize the primacy of participants’ contributions to this paper, as well as any contributions this paper may provide to future work. These limitations illuminate the need for greater justice and equity in community-based research. Only when individuals with lived experience are consistently, appropriately centered within all phases of research – design, data generation, and analysis—can any field of research truly represent what is studied.

## Conclusion

The testimonies of these participants demonstrate the many ways in which mental healthcare can become a means of recapitulating carceral goals when applied within the purview of the carceral state. Specifically, they highlight the ways in which such treatment is perceived to be punitive and controlling, rather than therapeutic and empowering. The act of bringing mental healthcare within the carceral state, an institution oriented toward control and punishment, fundamentally conflicts with core therapeutic principles of patient empowerment and agency. This is true regardless of whether the correct diagnosis is given or the correct treatment is administered. However, the patterns of diagnostic ambiguity and ill-fitting treatment described by participants illustrates how these changes may have functional ramifications, resulting in suboptimal care within the carceral state. Furthermore, the traumatic environment of the prison can exacerbate mental illness, create new trauma, and outweigh the benefits of any mandated treatment. Nevertheless, participants demonstrated a willingness to engage with mental healthcare systems in ways that were beneficial to them and that acknowledged mental healthcare as a distinct entity from the carceral system.

For many of the participants who contributed to this study, manifestations of mental illness created a catalyst for either imprisonment or escalation of confinement while in prison. Ultimately, treating symptoms of mental illness as meriting carceral or legal repercussions only serves to reinforce the criminalization of mental illness and the marginalization of those who live with it, particularly within communities of color. Furthermore, criminalizing mental illness draws attention away from the carceral state’s own role in creating mental illness. To be sure, developing collaborative strategies between criminal legal and mental health institutions and incorporating patient-centered care is an important stopgap measure in achieving ethical and equitable care for individuals with mental illnesses with criminal legal involvement. However, the ultimate goal must be to divest the ‘mental illness’ label of its implicit association with criminality entirely. As a society, we must strive to develop societal structures that reflect this by responding to mental illness not with imprisonment but with care, medical attention, and attention to structural factors contributing to mental illness, and to hold carceral systems accountable for the role they play in contributing to inequities in mental health and overall wellness.

## Data Availability

Not applicable.
